# Changes in Patients' Psychological Stress Before Surgery: An Observational Study Focusing on the Presence of Familiar Nurses in the Operating Room

**DOI:** 10.7759/cureus.92015

**Published:** 2025-09-10

**Authors:** Mayu Fukuda, Aoi Iwasa, Yusuke Mizuno, Tomoko Akase

**Affiliations:** 1 Department of Biological Science and Nursing/Perianesthesia Nursing, Yokohama City University, Graduate School of Medicine, Yokohama, JPN; 2 Department of Perianesthesia Nursing, Yokohama City University, Graduate School of Medicine, Yokohama, JPN; 3 Department of Anesthesiology, Yokohama City University Hospital, Yokohama, JPN

**Keywords:** autonomic nervous system, patient–nurse familiarity, preoperative anxiety, psychological stress, surgical nursing

## Abstract

Many surgical patients feel stressed and are exposed to the unusual environment of the operating room. Stressful conditions cause autonomic nervous system fluctuations and affect the body during and after surgery. Because familiar faces are believed to attract more attention and provide a sense of predictability and reassurance, thereby helping to reduce anxiety, the involvement of familiar nurses in the operating room may contribute to easing psychological stress. In this study, we aimed to observe changes in patients’ psychological stress before surgery, focusing on the presence or absence of familiar nurses in the operating room.

The subjects were patients undergoing surgery under general anesthesia. Of these 60 participants analyzed, 25 received a preoperative visit from the operating room nurse assigned to their surgery that day (acquaintance group), while 35 received a visit from a different operating room nurse (no-acquaintance group). To investigate the sequential changes in patients’ psychological stress during the preoperative period, autonomic nerve activity indexes (sympathetic nerve activity index LF/HF, parasympathetic nerve activity index HF) were measured using a wearable device when leaving the ward, entering the operating room, and when anesthesia was administered.

As a result, the LF/HF in the no-acquaintance group was highest upon entry into the operating room, with a median value of 3.47. During anesthesia induction, the LF/HF was significantly higher than at the time of departure from the ward (p < 0.01), but significantly lower than upon entry into the operating room (p = 0.02). The acquaintance group showed the highest LF/HF at the time of ward departure, with a median value of 4.87. In this group, the LF/HF significantly decreased during anesthesia induction compared to entry into the operating room (p = 0.02). Regarding HF, the no-acquaintance group exhibited the lowest HF upon entry into the operating room (median: 8.22), which significantly increased during anesthesia induction (p = 0.02). Conversely, in the acquaintance group, the lowest HF was observed at ward departure (median: 10.42), and the highest HF was recorded upon entry into the operating room (median: 11.78). HF was significantly higher at both entry into the operating room (p = 0.01) and anesthesia induction (p = 0.04) as compared to ward departure.

In this study, patients’ psychological stress was objectively assessed using a wearable device at three time points: ward departure, entry into the operating room, and anesthesia induction. The results suggest that regardless of the presence of familiar nurses, patients exhibited decreased sympathetic activity and increased parasympathetic activity during anesthesia induction, indicating a reduction in psychological stress.

## Introduction

Anxiety and stress are common reactions among patients undergoing surgery. Surgical patients often experience significant psychological stress, which can impact both their recovery and overall outcomes [1-3]. Elevated preoperative stress levels not only influence the postoperative period but are also associated with increased complications and delayed postoperative recovery [4]. The operating room is a markedly different environment from the familiar routines of daily life, making it a potential source of stress for patients undergoing surgery. The presence of unfamiliar medical equipment and personnel can heighten anxiety. In fact, Gürsoy et al. (2016) found that fear of the unknown, fear of anesthesia, and the operating room setting itself are significant stressors for surgical patients [5]. It has recently been shown that without appropriate nursing interventions to reduce anxiety, patients’ preoperative anxiety levels tend to rise as the time of surgery approaches, highlighting the need for nonpharmacological methods to alleviate this anxiety [6]. Although operating room staff other than anesthesiologists have traditionally had limited contact with patients before and after surgery, studies have shown that preoperative visits by operating room nurses can help reduce patients’ anxiety levels [7,8]. Because familiar faces are believed to attract more attention and provide a sense of predictability and reassurance, thereby helping to reduce anxiety [9], the involvement of familiar nurses in the operating room may contribute to easing both preoperative anxiety and psychological stress. In clinical practice, the operating room nurse is frequently the same nurse who conducts the preoperative visit. Therefore, this study aimed to clarify changes in patients’ psychological stress before surgery based on the presence or absence of a familiar nurse. Preoperative anxiety activates the body’s stress response, increasing levels of stress hormones such as cortisol and catecholamines and lowering patients’ pain thresholds during and after surgery [10]. It also triggers changes in biological responses regulated by the autonomic nervous system such as elevated heart rate and cortisol levels [11]. Therefore, this study also focused on patients’ biological responses in the operating room to assess their stress levels.

## Materials and methods

Study design

This was an observational study.

Participants

The study included patients undergoing scheduled surgery under general anesthesia at Yokohama City University Hospital. To minimize the confounding effects of diurnal variation in autonomic nervous activity, only patients entering the operating room between 8:30 and 9:00 am were included. The inclusion criteria were an age of ≥20 years, receipt of a verbal explanation of the study and provision of written consent, and the ability to walk independently into the operating room. The exclusion criteria were fragile skin or allergies to tape, plaster, or metal; entrance into the operating room via wheelchair or stretcher; presence of a cardiac pacemaker; current treatment with antianxiety or antidepressant medications; arrhythmia; inaccessible patient data; treatment with high-level thoracic epidural anesthesia (Th1-3); entry into the operating room at a time other than 8:30-9:00 am; and absence of a preoperative visit. Patients who received a preoperative visit from the same operating room nurse who would be present during surgery were defined as the acquaintance group. Those who received a preoperative visit from a different operating room nurse were defined as the no-acquaintance group.

Study period

The survey was conducted from March 2021 to August 2021.

Study procedure

Anesthesiology Outpatient Clinic

The researcher explained the study to the participants and provided a written explanation. After confirming that the participants fully understood the content, written consent for participation was obtained.

Day Before Surgery

The researcher gave each participant a written explanation of the procedure for the day of surgery.

Day of Surgery

On departure from the hospital ward: The researcher, who had provided the explanation the day before surgery, attached the wearable device to the participant’s anterior chest.

In the operating room (during anesthetic induction): After anesthesia was induced, the wearable device was removed from the participant’s anterior chest.

Survey items: 1) Background of the participants - Information on sex, age, surgical history, marital status, presence of family members living with the patient, presence of malignant disease, anesthesia method, planned surgical procedure, and medical department was collected from the electronic medical record. Planned surgical procedures were classified into three categories: large, medium, or small, based on the classification of surgical invasiveness described in the National Institute for Health and Care Excellence guideline titled Routine Preoperative Tests for Elective Surgery. The presence or absence of prior acquaintance with the anesthesiologist in charge of the surgery was confirmed directly with the anesthesiologist on the day of surgery by the researcher or a research associate; 2) Autonomic nerve activity indices - The outcome measures in this study were indices of sympathetic nerve activity (ratio of LF component to HF) and parasympathetic nerve activity (HF) were assessed at three time points: departure from the ward, entry into the operating room, and induction of anesthesia. Cardiac potentials and pulse waves were measured by attaching a Silmee™ Bar Type Lite (TDK Corporation, Tokyo, Japan) (hereafter referred to as the “wearable device”) to the anterior chest of each participant--specifically, three lateral finger-widths below the collarbone. The device was configured according to the recommended settings for estimating autonomic balance during the daytime: cardiac potential sampling cycle of 1000 Hz, pulse sampling cycle of 125 Hz, acceleration sampling cycle of 125 Hz, lower heart rate cycle limit of 444 ms, upper limit of 1715 ms, and both heart rate and pulse rate detection thresholds set at 40-150 bpm. Heart rate variability analysis was performed using the Small System Application, which is compatible with the Silmee™ Bar Type Lite. This application automatically calculated the HF (0.15-0.50 Hz), LF (0.04-0.15 Hz), and LF/HF ratio every 60 seconds from the R-R interval time series data. The LF/HF ratio and HF, as indices of autonomic nervous activity, were measured at three time points: at ward departure (values were averaged over a two-minute period, beginning five minutes after the wearable device was attached, while the participant was seated at the edge of the bed), at operating room entry (values were averaged over two minutes starting from the moment the participant, in a standing position, met the nurse in charge in the operating room), and at induction of anesthesia (values were averaged over two minutes immediately before the administration of intravenous anesthesia, just prior to the participant being placed in a supine position and oxygenation being initiated). At all time points, there were no changes in posture during measurement.

Analytical methods

Statistical analysis was performed using SPSS version 27 (IBM Corp., Armonk, NY, USA), with a significance level set at 5%.

Patients’ Basic Attributes

Comparisons between the acquaintance and no-acquaintance groups were conducted using Pearson’s chi-square test or Fisher’s exact probability test for categorical variables: sex, surgical history, marital status, presence of family members living with the patient, presence of malignant disease, degree of surgical invasiveness, anesthetic method, prior acquaintance with the anesthesiologist in charge of surgery, and medical department. For age, the Shapiro-Wilk test was used to assess normality, followed by analysis with the Mann-Whitney U test.

LF/HF Ratio and HF

Comparisons of the LF/HF ratio and HF across all time points within each group were conducted using post hoc tests with Bonferroni correction, based on significant results obtained from the Friedman test.

Ethical considerations

This study was approved and conducted in accordance with the guidelines of the Yokohama City University Research Ethics Committee for Life Sciences and Medicine involving human subjects (Approval No. B201200003).

## Results

Patient selection

**Figure 1:**
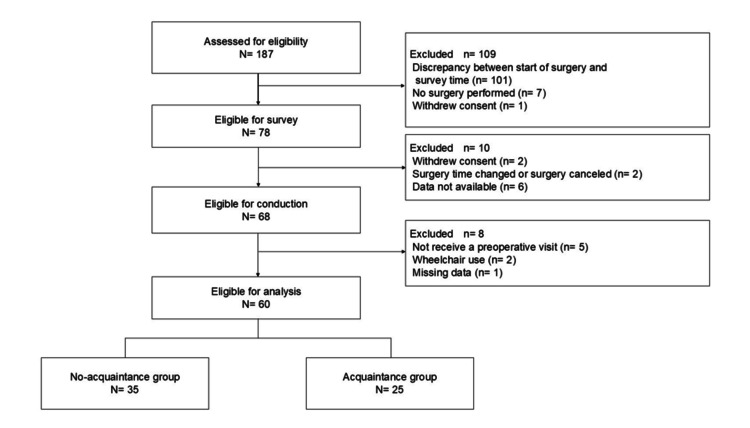
Flow chart of patient selection

The selection process for the study participants is illustrated in Figure 1. A total of 187 individuals who met the inclusion criteria provided consent during their preoperative outpatient visit. Of these, 101 were excluded because the start time of their surgery did not match the time of the investigation, 7 because they did not undergo surgery during the study period, and 1 because they withdrew consent, resulting in 78 individuals eligible for inclusion. Among these 78 patients, 2 withdrew consent the day before the survey, 2 had their surgery time changed or surgery canceled, and data were not available for 6, leaving 68 participants. Of these, 5 because they did not receive a preoperative visit, 2 because they entered the operating room in a wheelchair, and 1 because of missing data, resulting in 60 participants eligible for analysis. Of these 60 participants analyzed, 25 received a preoperative visit from the operating room nurse assigned to their surgery that day (acquaintance group), while 35 received a visit from a different operating room nurse (no-acquaintance group).

Patients’ background

The basic attributes of the two groups are presented in Table 1. No significant differences in any variables were observed between the groups, except for the number of participants in each group.

**Table 1:** Autonomic nerve activity at ward departure, operating room entry, and anesthetic induction Data are shown as the number (%) or median (interquartile range). ^a^X^2^test; ^b^Mann-Whitney U test; ^c^Fisher's exact test

Attribute		No-acquaintance (n=35)	Acquaintance (n=25)	P-value	X^2^-value	U-value	N=60
Sex	Male	18 (51.4)	10 (40.0)	0.38	0.8	-	^a^
	Female	17 (48.6)	15 (60.0)	-	-	-	
Age (years)	-	62.0 (49.0 - 73.0)	54.0 (38.0 - 73.0)	0.25	-	515	^b^
Surgical History	Yes	19 (54.3)	9(36.0)	0.16	1.96	-	^a^
	No	16 (45.7)	16 (64.0)	-	-	-	-
Marriage	Married	24 (68.6)	18 (72.0)	0.78	0.08		^a^
	Single	11 (31.4)	7 (28.0)	-	-	-	-
Cohabiting Family	Yes	27 (77.1)	21 (84.0)	0.38	-	-	^c^
	None	8 (22.9)	4 (16.0)	-	-	-	-
Malignant Disease	Diagnosed	18 (51.4)	12 (48.0)	0.76	0.55		^a^
	Suspicious	7 (20.0)	7 (28.0)	-	-	-	-
	None	10 (28.6)	6 (24.0)	-	-	-	-
Surgical Invasion	Large	28 (80.0)	15 (60.0)	0.09	2.87		^a^
	Medium	7 (20.0)	10 (40.0)	-	-	-	-
	Small	0 (0.0)	0 (0.0)	-	-	-	-
Anesthesia	General, General and Epidural	22 (62.8)	17 (68.0)	0.68	0.17		^a^
	General and Nerve block	13 (37.1)	8 (32.0)	-	-	-	-
Prior Contact With Anesthesiologist	Yes	33 (94.3)	20 (80.0)	0.1	-	-	^c^
	No	2 (5.7)	5 (20.0)				
Clinical Department	Gastrointestinal Surgery	8 (22.9)	4 (16.0)	0.61	6.83	-	^a^
	Gynecology	6 (7.1)	8 (32.0)	-	-	-	-
	Urology	6 (17.1)	4 (16.0)	-	-	-	-
	Neurological Surgery	5 (14.3)	1 (4.0)	-	-	-	-
	Orthopedic Surgery	2 (5.7)	4 (16.0)	-	-	-	-
	Otorhinolaryngology	2 (5.7)	1 (4.0)	-	-	-	-
	Dermatology	1 (2.9)	0 (0.0)	-	-	-	-
	Oral Surgery	1(2.9)	0 (0.0)	-	-	-	-
	Breast and Thyroid Gland Surgery	3 (8.6)	3 (12.0)	-	-	-	-
	Plastic Surgery	1 (2.9)	0 (0.0)	-	-	-	-

Autonomic nervous system activity at three time points

**Figure 2:**
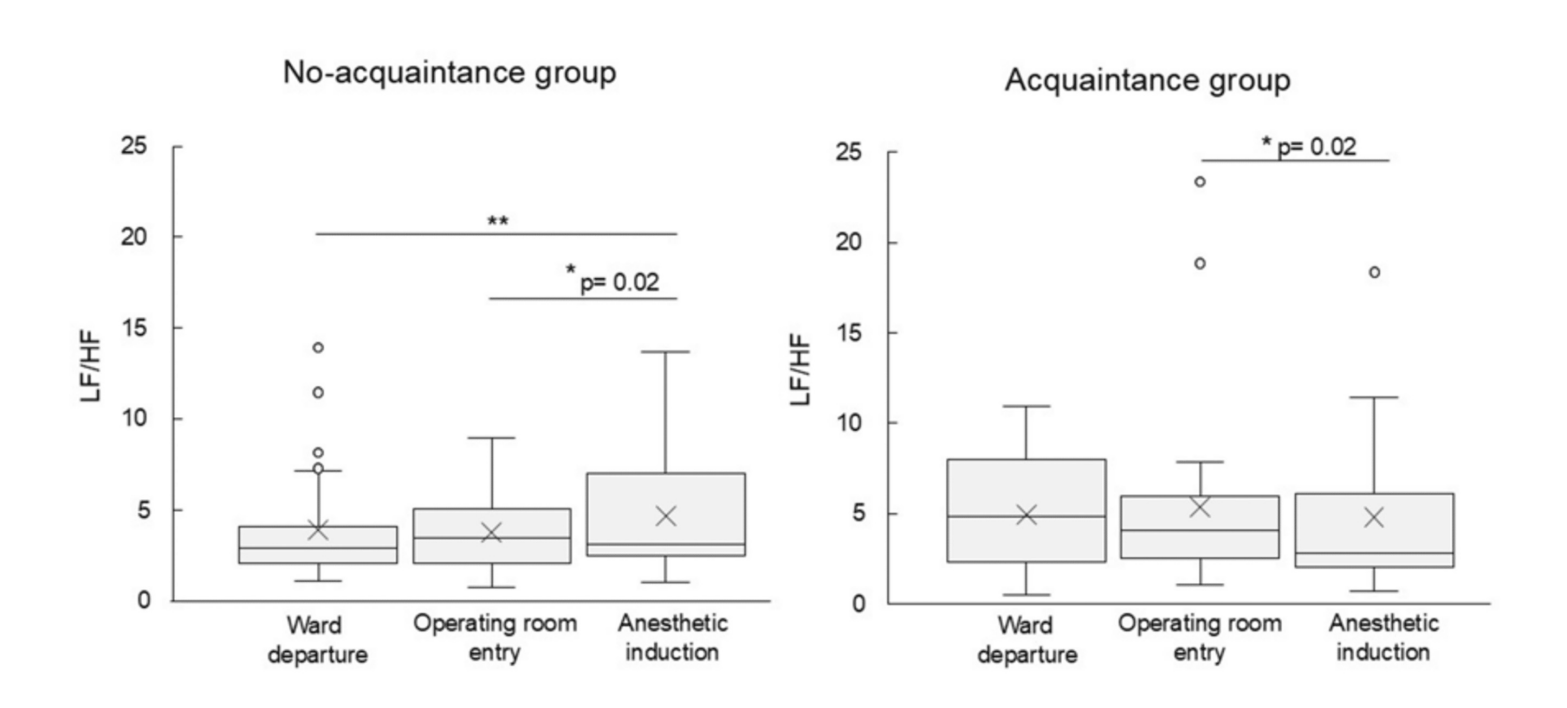
Transition of LF/HF ratio across three perioperative time points: ward departure, operating room entry, and anesthetic induction All comparisons were performed using post hoc tests with Bonferroni correction following the Friedman test. **p<0.01, *p<0.05

The LF/HF ratios in the two groups were compared at three time points: ward departure, operating room entry, and anesthetic induction (Figure 2). The no-acquaintance nurse group showed the LF/HF of 2.90 (IQR: 2.07-4.13) at ward departure, 3.47 (IQR: 2.08-5.09) at operating room entry, and 3.15 (IQR: 2.45-7.03) during anesthesia induction. The highest value was observed at the operating room entry. The LF/HF during anesthesia induction was significantly higher than at ward departure (p < 0.01), while it was significantly lower than at operating room entry (p = 0.02).

In contrast, in the acquaintance group, the LF/HF was 4.87 (IQR: 2.37-8.05) at ward departure, 4.10 (IQR: 2.59-6.01) at operating room entry, and 2.88 (IQR: 2.04-6.16) during anesthesia induction, with the highest value observed at ward departure. The LF/HF significantly decreased during anesthesia induction compared to operating room entry (p = 0.02).

**Figure 3:**
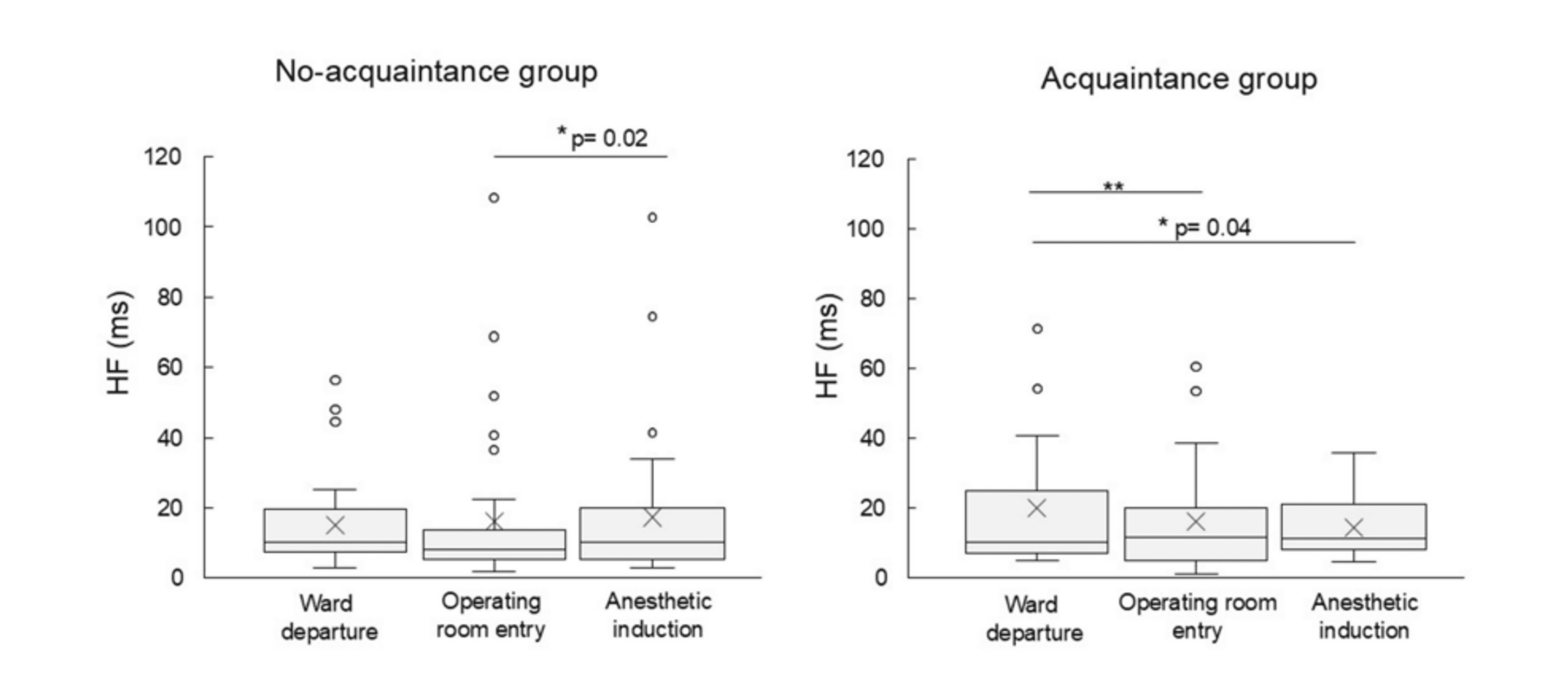
Transition of HF component across three perioperative time points: ward departure, operating room entry, and anesthetic induction All comparisons were performed using post hoc tests with Bonferroni correction following the Friedman test. The unit of HF is ms. **p<0.01, *p<0.05

Within each group, HF were compared at three distinct time points: departure from the ward, entry into the operating room, and induction of anesthesia (Figure 3). In the no-acquaintance group, the HF component was 10.12 (IQR: 7.41-19.52) ms at ward departure, 8.22 (IQR: 5.27-13.84) ms at operating room entry, and 10.14 (IQR: 5.45-19.79) ms during anesthesia induction. The lowest HF was observed at the operating room entry. A statistically significant increase in HF was observed during anesthesia induction compared to operating room entry (p = 0.02). In the acquaintance group, the HF were 10.42 (IQR: 7.27-25.05) ms at ward departure, 11.78 (IQR: 4.91-19.97) ms at operating room entry, and 11.27 (IQR: 8.13-20.99) ms during anesthesia induction. The lowest value was recorded at ward departure, while the highest was at operating room entry. The HF at both operating room entry (p = 0.01) and anesthesia induction (p = 0.04) were significantly higher than those at ward departure.

## Discussion

This observational study objectively revealed temporal changes in preoperative psychological stress from ward departure to anesthesia induction using a biometric device--an approach that had not been achieved through subjective measures. In some clinical settings, nurses who conduct preoperative visits are also present in the operating room. Based on this context, the study monitored psychological stress responses in patients with and without the presence of an acquaintance nurse. Regardless of the nurse’s familiarity, patients exhibited decreased sympathetic nervous activity and increased parasympathetic nervous activity during anesthesia induction. These physiological responses suggest that patients were in a more relaxed state at the time of anesthesia induction, indicating a reduction in psychological stress.

Changes in the LF/HF at ward departure, operating room entry, and anesthesia induction

In the no-acquaintance group, the LF/HF peaked at the time of operating room entry and significantly decreased during anesthesia induction. At all three time points, the LF/HF remained around 3. In contrast, the acquaintance group showed the highest LF/HF at ward departure (4.89), followed by a decrease at operating room entry (4.10) and further at anesthesia induction (2.88). Generally, a higher LF/HF indicates a state of sympathetic dominance. The elevated LF/HF at ward departure in the acquaintance nurse group suggests that these patients were already in a state of heightened sympathetic nervous activity at that time. One possible explanation for this observation is that the proportion of women was 48.6% in the no-acquaintance group and 60.0% in the acquaintance group. Because women are more likely to experience preoperative anxiety than men [12], Anxiety is known to activate sympathetic nervous activity. Therefore, the acquaintance group--having a higher proportion of women--was considered to represent a more anxious population. Furthermore, the number of gynecology patients was higher in the acquaintance group than in the no-acquaintance group. A previous study reported that anxiety in surgical patients peaks prior to anesthesia [12]. However, in the present study, the peak LF/HF was observed at the operating room entry in the no-acquaintance group and at ward departure in the acquaintance group, rather than immediately before anesthesia induction. In fact, in both groups, the LF/HF significantly decreased from operating room entry to anesthesia induction. Interestingly, in the no-acquaintance group, the LF/HF at anesthesia induction was significantly higher than at ward departure. This finding suggests that, at least in this group, patients may have been in a more sympathetic-dominant state during anesthesia induction compared to ward departure, indicating a possible increase in psychological stress as the procedure progressed.

Changes in the HF at ward departure, operating room entry, and anesthesia induction

Surgical stress and invasive procedures have been reported to increase sympathetic nervous system activity and decrease parasympathetic activity [13,14]. In the present study, HF in the no-acquaintance group significantly increased from operating room entry to anesthesia induction. In the acquaintance group, HF significantly increased from ward departure to operating room entry, and also from ward departure to anesthesia induction. The key contextual difference between the two groups was whether patients encountered an acquaintance nurse upon entering the operating room. The earlier increase in HF observed in the acquaintance group at the time of operating room entry may be associated with the presence of a familiar nurse, potentially contributing to enhanced parasympathetic activity at an earlier stage.

Limitations

This study has several important limitations. First, psychological states, such as personality traits and levels of preoperative anxiety, vary among individuals and may have influenced the results. For example, in terms of LF/HF, the acquaintance group exhibited a more sympathetic-dominant state at ward departure compared to the no-acquaintance group. In addition, individual factors, such as participants’ educational background, knowledge regarding surgical procedures and analgesia, and history of previous surgeries, may also have affected the outcomes. However, this study did not incorporate subjective measures or conduct multifaceted analyses combining physiological and psychological data. Additionally, as an observational study, no stratification of participants was performed, which should be taken into account when interpreting the findings. Second, the study was conducted at a single institution, which limits the generalizability of the results to other clinical settings.

## Conclusions

Psychological stress in surgical patients was objectively assessed from ward departure to anesthesia induction through the application of a wearable device capable of measuring autonomic nervous system activity. The results demonstrated that, irrespective of the presence of an acquaintance nurse in the operating room, patients exhibited reduced psychological stress at the time of anesthesia induction--a phase typically characterized by elevated anxiety. These findings indicate that patients enter anesthesia in a more physiologically relaxed state than previously assumed.
